# The multifarious roles of Tol-Pal in Gram-negative bacteria

**DOI:** 10.1093/femsre/fuaa018

**Published:** 2020-05-30

**Authors:** Joanna Szczepaniak, Cara Press, Colin Kleanthous

**Affiliations:** Department of Biochemistry, South Parks Road, University of Oxford, Oxford OX1 3QU, UK; Department of Biochemistry, South Parks Road, University of Oxford, Oxford OX1 3QU, UK; Department of Biochemistry, South Parks Road, University of Oxford, Oxford OX1 3QU, UK

**Keywords:** cell envelope, outer membrane, peptidoglycan, divisome, proton motive force, Ton

## Abstract

In the 1960s several groups reported the isolation and preliminary genetic mapping of *Escherichia coli* strains tolerant towards the action of colicins. These pioneering studies kick-started two new fields in bacteriology; one centred on how bacteriocins like colicins exploit the Tol (or more commonly Tol-Pal) system to kill bacteria, the other on the physiological role of this cell envelope-spanning assembly. The following half century has seen significant advances in the first of these fields whereas the second has remained elusive, until recently. Here, we review work that begins to shed light on Tol-Pal function in Gram-negative bacteria. What emerges from these studies is that Tol-Pal is an energised system with fundamental, interlinked roles in cell division – coordinating the re-structuring of peptidoglycan at division sites and stabilising the connection between the outer membrane and underlying cell wall. This latter role is achieved by Tol-Pal exploiting the proton motive force to catalyse the accumulation of the outer membrane peptidoglycan associated lipoprotein Pal at division sites while simultaneously mobilising Pal molecules from around the cell. These studies begin to explain the diverse phenotypic outcomes of *tol-pal* mutations, point to other cell envelope roles Tol-Pal may have and raise many new questions.

## Teaser

The trans-envelope Tol-Pal assembly has at least two, recently defined, interconnected roles during cell division in bacteria; stabilising the outer membrane by actively depositing the peptidoglycan-binding lipoprotein Pal at division sites and orchestrating local structural changes in the peptidoglycan.

## INTRODUCTION & BACKGROUND

The cell envelope of Gram-negative bacteria is comprised of a symmetric inner membrane and an asymmetric outer membrane with an intervening layer of peptidoglycan (PG) in the periplasm. The outer membrane is a robust protective barrier that shields the bacterium from the immune system and excludes major classes of antibiotics such as vancomycin thereby contributing to multidrug resistance. The outer membrane is not energised and there is no ATP in the periplasm so active processes must be coupled either to ATP hydrolysis in the cytoplasm or the proton motive force (PMF) across the inner membrane. The Tol-Pal system straddles the three layers of the cell envelope, is coupled to the PMF and plays a major role in constricting the outer membrane (Egan 2018).

*tol* (*tol-pal*) genes were originally identified through mutations that engendered *Escherichia coli* tolerance towards colicins and bacteriophages (Gratia 1964; Reeves 1966; Hill and Holland 1967; Nagel de Zwaig and Luria 1967). The *tol-pal* operon in *E. coli* is composed of seven genes, five of which are generally regarded as comprising the core Tol-Pal system in bacteria and in the following order: *tolQ*, *tolR*, *tolA*, *tolB* and *pal*. Deletion of these core genes generates the classical *tol* phenotype of outer membrane instability (see below) and all, with the exception of *pal*, also result in tolerance towards group A colicins and filamentous bacteriophages. Colicins are *E. coli*-specific, multidomain bacteriocins that harness the PMF through the Tol-Pal or ExbB-ExbD-TonB (Ton) systems to promote translocation of their cytotoxic domains across the OM (Cascales *et al*. 2007; Kleanthous 2010). Phages also exploit these systems and appear to use similar strategies to colicins to deliver epitope signals into the cell (Jakes, Davis and Zinder 1988; Riechmann and Holliger 1997). The likely reason *E. coli* Pal is not targeted by colicins is because it is not coupled to the PMF, which is needed for outer membrane translocation (Jetten and Jetten 1975; Hancock and Braun 1976; Lieberman and Hong 1976; Braun and Herrmann 1993). The other genes that are part of the *tol-pal* operon, but which do not yield the same phenotypes as *tol-pal* mutations, are *ybgC*, a cytoplasmic lipid thioesterase, and *cpoB/ybgF*, a periplasmic regulator of peptidoglycan (PG) peptide crosslinking. The majority of this review is focused on core Tol-Pal proteins but additional components are included where their functions intersect with those of the Tol-Pal assembly.

The *tol-pal* operon is found in all subclasses of proteobacteria and prominent in other phyla, principally the Chlorobi, Chlamydiae and Acidobacteria (Krachler *et al*. 2010b). With the exception of *ybgC* and *cpoB/ybgF*, which are sometimes absent or replaced by other genes, the order of *tol-pal* genes is also highly conserved (Sturgis 2001). The essentiality of *tol-pal* genes varies in different species; the operon is not essential in *E. coli* K-12 but is essential in *Caulobacter crescentus (Yeh et al*. 2010*)* and *Pseudomonas aeruginosa* (Dennis, Lafontaine and Sokol 1996; Lo Sciuto *et al*. 2014). In *P. aeruginosa*, *tol-pal* expression is modulated by iron in the medium and the growth phase of the organism (Lafontaine and Sokol 1998; Duan *et al*. 2000). Beyond early studies suggesting that *tol-pal* expression is induced by RcsC in *E.coli* (Clavel *et al*. 1996) and quantitative proteomics studies showing all the components are expressed in both rich and defined media (Li *et al*. 2014) surprisingly little is known about how the system is regulated.

The pleiotropic outer membrane instability phenotype typically associated with *tol-pal* mutations has been well-characterised but remains poorly understood, primarily because of the difficulties in differentiating traits attributable directly to *tol-pal* genes from those that are downstream effects, such as the activation of cell envelope stress responses. Lopes, Gottfried and Rothfield (1972) first characterised ‘leaky’ (*lky*) mutants in *E. coli* and *Salmonella typhimurium* (Lopes, Gottfried and Rothfield 1972) that were later mapped to the *tol-pal* operon (Lazzaroni and Portalier 1981). *lky* cells have a permeabilised outer membrane that releases ribonuclease I from the periplasm. In 1976, Weigand and Rothfield demonstrated that *Salmonella* cells with a standard *lky* mutant phenotype display a defect in outer membrane invagination during formation of the septum. Using electron microscopy, they showed ‘ballooning’ of the outer membrane from the septal region, with the formation of the large bleb on the surface of the cell (Weigand and Rothfield 1976). *tol-pal* cells bleb during division and produce copious outer membrane vesicles (OMVs). Indeed, *tolR* mutants are used to increase yields of OMVs for the production of vaccines against nontyphoidal *Salmonella* (Micoli *et al*. 2018) and *Shigella flexneri* (Pastor *et al*. 2018).

*E. coli* K-12 *tol-pal* strains grow normally at 37°C in high salt growth media, but produce mucoid colonies at 30°C and are not viable at 42°C (Nomura and Witten 1967; Bernstein, Rolfe and Onodera 1972; Yakhnina and Bernhardt 2020). In LB media lacking salts at 30°C, *E. coli tol-pal* cells filament (Gerding *et al*. 2007). Conversely, *E. coli tolA* mutants grow in chains in both high and low osmolarity media (Meury and Devilliers 1999) and are unable to grow at high hydrostatic pressure (Black *et al*. 2013). *tol*-*pal* deficient cells tend to have increased sensitivity to surface active compounds such as bile salts (for example, deoxycholic acid), detergents such as SDS (Nagel de Zwaig and Luria 1967) and drugs such as polymyxin B (Lazdunski and Shapiro 1972). *tol-pal* cells are also more sensitive to *β*-lactam antibiotics (Davies and Reeves 1975), and susceptible to vancomycin (Onodera, Rolfe and Bernstein 1970) and novobiocin (Foulds and Barrett 1973), phenotypes that are consistent with the barrier function of the outer membrane being compromised.

Another well-documented effect of *tol-pal* mutations is their impact on the ratio of phospholipids-to-lipopolysaccharide (LPS) in the outer membrane (Shrivastava, Jiang and Chng 2017; Masilamani, Cian and Dalebroux 2018). *tolA* cells are unable to express full O-antigen on their surface (Gaspar *et al*. 2000; Vines *et al*. 2005), probably due to a shortened core LPS (Anderson, Wilson and Oxender 1979). When the O-antigen is shortened (Rottem and Leive 1977) or the ratio of phospholipids-to-LPS is increased, the outer membrane becomes more fluid and thus more susceptible to mechanical stress as suggested by course-grain simulations (Jefferies, Shearer and Khalid 2019).

*tol-pal* mutations activate two main cell envelope stress response pathways, Rcs and σ^E^. The Rcs pathway senses lateral interactions between LPS molecules (Konovalova, Mitchell and Silhavy 2016) and modulates the expression of genes responsible for production of biofilm, capsule or modification of lipids (Wall, Majdalani and Gottesman 2018). Clavel *et al*. (1996) demonstrated that a mutation in *E. coli* RcsC increases its kinase activity and downregulates *tolQRA* expression (Clavel *et al*. 1996). When *tolA* (Clavel *et al*. 1996) or *tolB* (Mouslim, Latifi and Groisman 2003) genes are deleted, cells upregulate capsule production in a RcsC-RcsB-dependent manner, resulting in mucoid colonies at low temperatures (Bernstein, Rolfe and Onodera 1972). Similar activation of the Rcs pathway is seen in *S. typhimurium tol-pal* mutants (Masilamani, Cian and Dalebroux 2018). The σ^E^ stress response is activated by both misfolded outer membrane proteins and LPS that is retained in the periplasm (Lima *et al*. 2013), resulting in transcription of genes involved in outer membrane protein folding (the Bam complex) and degradation of misfolded proteins (DegP) (for a review of stress systems see (Mitchell and Silhavy 2019). Vines *et al*. (2005) demonstrated that both *tolA* and *pal* mutants increase their expression of *degP (**Vines et al. 2005**)*, consistent with increased outer membrane fluidity and problems with outer membrane protein insertion (Storek *et al*. 2019).

Tol-Pal is required for pathogenesis and virulence in many species of Gram-negative bacteria, including uropathogenic *E. coli* (Hirakawa *et al*. 2019), *Edwardsiella ictaluri* (Abdelhamed *et al*. 2016), *Salmonella typhimurium* (Bowe *et al*. 1998), *Erwinia chrysanthemi* (Dubuisson *et al*. 2005) and *Haemophilus ducreyi* (Fortney *et al*. 2000). *Pseudomonas putida tolB* cells are less efficient at forming biofilms (Lopez-Sanchez *et al*. 2016) with similar phenotypes reported for *Burkholderia pseudomallei tolB* (Khan *et al*. 2019) and *E. coli tolA* cells (Ranjith *et al*. 2019). Indeed, in *P. aeruginosa* and *Xylella fastidiosa*, *tol-pal* genes are overexpressed during biofilm formation (Whiteley *et al*. 2001; Santos *et al*. 2015). Pal has been reported to be essential for persister cell survival during antibiotic treatment of *E. coli* (Sulaiman, Hao and Lam 2018). Uropathogenic *E. coli pal-*deficient cells are unable to produce capsule and are sensitive to serum (Diao *et al*. 2017). Finally, an aspect of Pal biology that is not well-understood is its apparent dual orientation in the outer membrane of some species, which has been exploited to produce vaccines. Pal is an abundant lipoprotein, normally inserted in the inner leaflet of the outer membrane by the Lol system (Ichihara, Hussain and Mizushima 1981). However, some Pal molecules are seemingly exposed on the surface of bacteria. This feature has enabled Pal-directed vaccines to be developed against *Haemophilus influenzae* (McMahon *et al*. 2005), *Legionella pneumophila* (Mobarez *et al*. 2019) and *Acinetobacter baumannii* (Lei *et al.*^2019^).

## STRUCTURE AND FUNCTION OF CORE Tol-Pal PROTEINS

**Figure 1.**
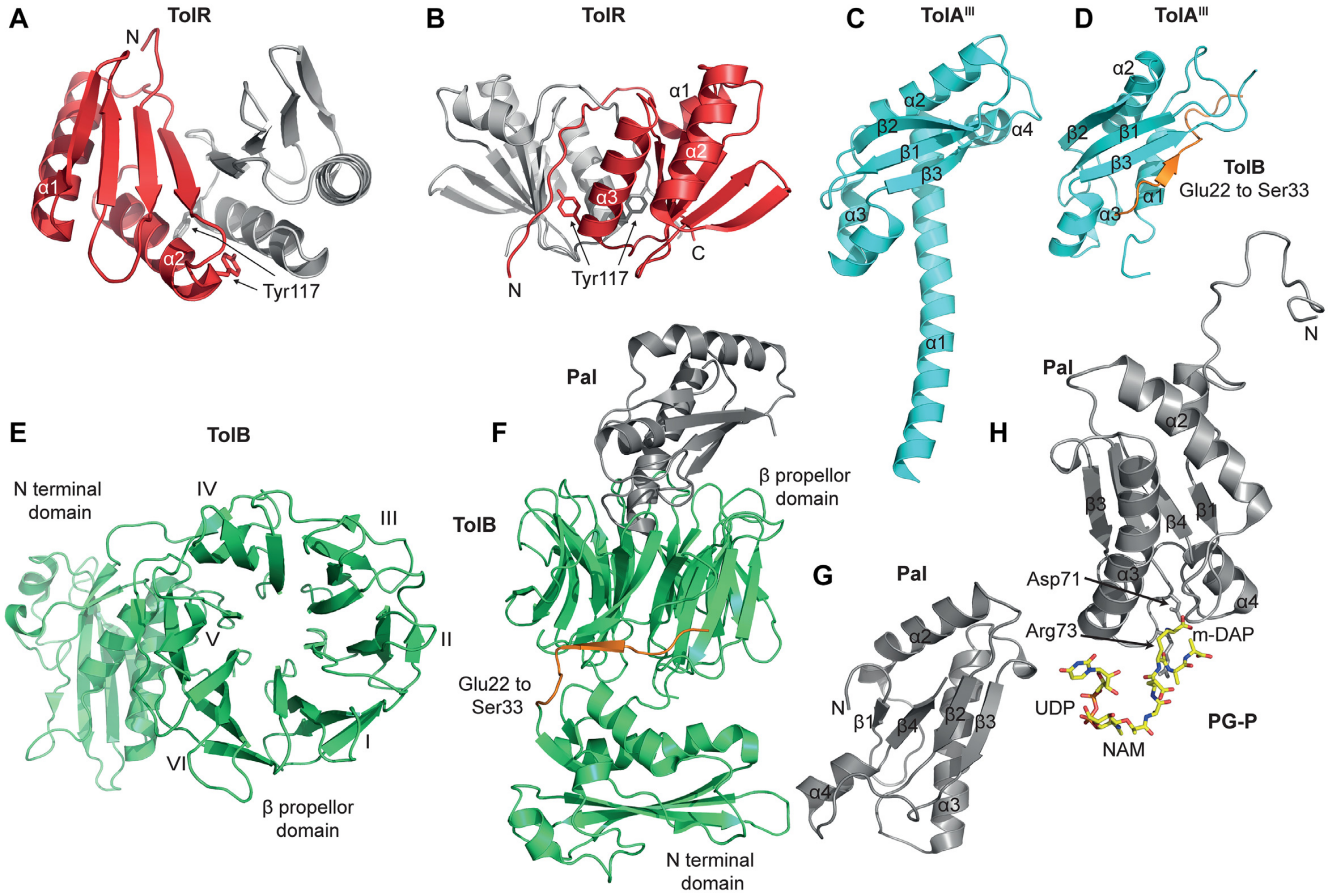
Structures of Tol-Pal proteins. The figure presents all currently known structures in the PDB for soluble domains and/or complexes of Tol-Pal proteins. **A**, The solution-state structure of the TolR periplasmic domain dimer in its ‘open’ PG-binding conformation (PDB code: 2JWK); the groove running between the two monomers is thought to be the PG binding site. The structure is that of *H. influenzae* TolR (residues 59–130) (Parsons, Grishaev and Bax 2008). See text for details. **B**, Crystal structure of the strand-swapped TolR periplasmic domain dimer, the ‘closed’ state (PDB code: 5BY4). This is the *E. coli* TolR structure (residues 36–142) in which the additional N- and C-terminal sequences occlude the deep groove between the monomers and block binding to PG (Wojdyla *et al*. 2015). In both *a* and *b*, the position of Tyr117 is shown. A Tyr117Cys substitution forms a spontaneous disulphide bond between TolR monomers that inactivates the Tol-Pal system *in vivo* (Goemaere *et al*. 2007b). These residues are only close enough to form a disulphide in *b* suggesting inactivation comes from stabilising the closed state of the stator complex (Wojdyla *et al*. 2015). **C**, Crystal structure of *P. aeruginosa* TolA^III^ (PDB code: 1LR0) (Witty *et al*. 2002). **D**, Solution state structure of the *P. aeruginosa* TolA^III^-TolB^22–33^ complex (PDB code: 6S3W). TolB binds through a β-strand augmentation mechanism in which the C-terminal α-helix (α4) of TolA is displaced by the N-terminus of TolB (Glu22-Ser33, in orange) (Szczepaniak *et al*. 2020). **E**, Crystal structure of *E. coli* TolB (PDB code: 1CRZ). TolB is comprised of an N-terminal α/β domain and a six-bladed β-propeller domain (Abergel *et al*. 1999). **F**, Crystal structure of the *E. coli* TolB-Pal complex (PDB code:2W8B) (Bonsor *et al*. 2009). The structure is rotated 90° relative to TolB in *e*. Shown in orange (Glu22-Ser33) is the N-terminus of TolB that becomes ordered in the Pal-bound state (Bonsor *et al*. 2009). **G**, Crystal structure of *E. coli* Pal (1OAP). **H**, Solution state structure of *H. influenzae* Pal bound to the peptidoglycan precursor UDP-N-acetylmuramyl-L-Ala-α-D-Glu-*m*-Dap-D-Ala-D-Ala (PDB code: 2AIZ) (Parsons, Lin and Orban 2006). The figure shows how the *m-*DAP residue of PG reaches into the binding pocket of Pal.

In order to understand the principal functions of the Tol-Pal system a complete picture of its structural biochemistry is needed to which specific cellular phenotypes can be linked. The following sections summarise what is known of the structure and the function of core components of the Tol-Pal system; TolQ, TolR, TolA in the inner membrane, TolB in the periplasm and Pal in the outer membrane. While some structures are known (Fig. 1) others, such as the TolQ-TolR-TolA complex, are not. In these cases, inferences are made from past mutational, biochemical and *in vivo* studies along with functional similarities to homologous systems, primarily the ExbB-ExbD-TonB and MotA-MotB assemblies in Gram-negative bacteria.

All three systems are PMF-linked nanomachines that drive mechanical processes at or beyond the outer membrane of Gram-negative bacteria and have related inner membrane stator complexes. The MotA-MotB complex drives rotation of the bacterial flagellum (Berg 2003), ExbB-ExbD powers TonB-mediated nutrient uptake through outer membrane transporters (Noinaj *et al*. 2010) and TolQ-TolR energises TolA to dissociate TolB-Pal complexes at the outer membrane (Szczepaniak *et al*. 2020). These simple comparisons emphasise three important points. First, that related stators can have very different biological functions. Second, that the three stator complexes have conserved residues implicated in proton transfer and almost certainly share common folds. Third, that the mechanical mechanism used by these stators to generate PMF-induced force is likely to be common to all of them, and indeed shared with other stators such as that used to drive gliding motility in *Myxococcus xanthus* (Youderian *et al*. 2003).

Here, we focus on Tol-Pal but reference other systems where they illuminate aspects of Tol-Pal structure-function. Before detailing the structural biochemistry of each Tol-Pal component we briefly summarise current understanding of the biological function of Tol-Pal. Tol-Pal's PMF-mediated disruption of TolB-Pal complexes in the outer membrane is the means by which the system accumulates Pal at the division site. TolB and Pal are unique to the Tol-Pal system. Pal binds the cell wall so by increasing its concentration at the division site the cell has a way of preventing the ballooning associated with *tol-pal* mutations. TolB is the means by which the PMF-linked inner membrane stator ensures Pal is displaced wherever the stator is located, which, as we shall see, is linked to formation of the division septum.

### The TolQ-TolR stator complex

TolQ is 25-kDa protein composed of three transmembrane helices (Kampfenkel and Braun 1993; Vianney *et al*. 1994). Although little structural or biochemical data are available for TolQ a number of mutational and crosslinking studies have established connections to partner proteins TolR and TolA (Derouiche *et al*. 1995; Lazzaroni *et al*. 1995; Germon *et al*. 1998; Journet *et al*. 1999; Zhang *et al*. 2011). Given the paucity of structural information we base the following on recent studies of the ExbB-ExbD complex. TolQ and TolR are homologues of ExbB and ExbD, with 35% and 29% sequence identity, respectively, between each homologue. Importantly, overexpression of *tolQ-tolR* complements a strain in which *exbB* and *exbD* are deleted, and vice versa, hinting at a common mechanism (Braun and Herrmann 1993). Six structures of ExbB, some in complex with ExbD, have been published often with differing subunit stoichiometry (Celia *et al*. 2016; Maki-Yonekura *et al*. 2018; Celia *et al*. 2019). ExbB is comprised of seven α helices, three of which are transmembrane (α2, α6 and α7). The transmembrane helices of individual ExbB molecules extend into the cytoplasm where they contribute to five-helix bundles that combine to form a chamber (Celia *et al*. 2016). In the pentameric structures of ExbB the chamber is a closed cavity with five-fold symmetry (Celia *et al*. 2016). A hexameric structure of the ExbB-ExbD complex has also been reported, when crystallised at high pH (pH 9.0) (Maki-Yonekura *et al*. 2018), but how relevant this structure is to the *in vivo* functioning of the complex is unclear.

**Figure 2.**
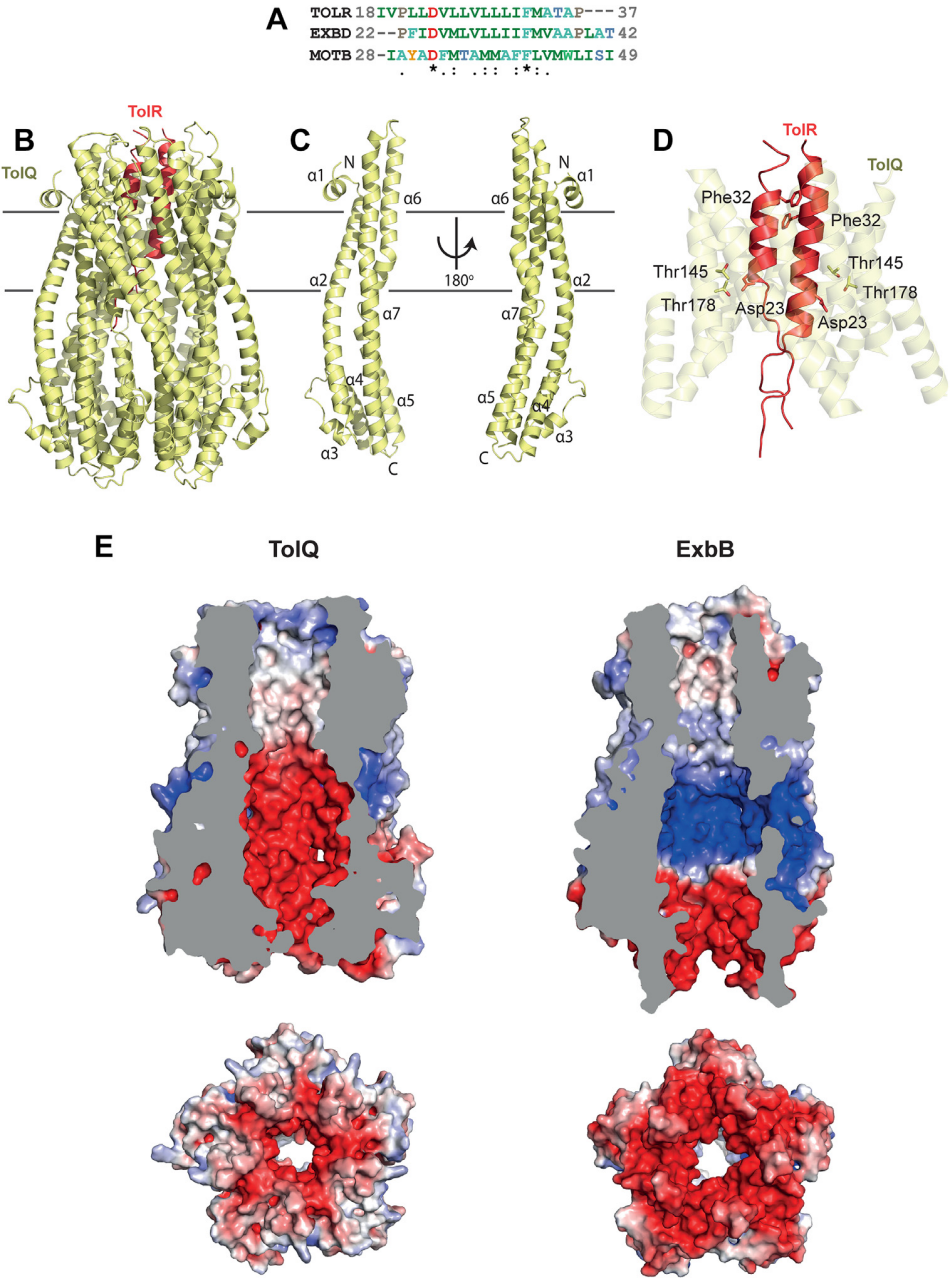
Model of the TolQ-TolR stator. **A**, Alignment of the trans-pore helix regions of *E. coli* TolR, ExbD and MotB. Asp23 and Phe32 (TolR numbering) are conserved across all three proteins. The alignment was generated using MUSCLE ClustalW. **B**, Model of the TolQ-TolR complex based upon the 5:2 structure of ExbB-ExbD (Celia *et al*. 2019). Horizontal lines represent approximate position of the inner membrane. The model was generated using SWISS-MODEL (Waterhouse *et al*. 2018) (https://swissmodel.expasy.org/). **C**, Model of each TolQ monomer. **D**, Co-localization of functionally important TolQ and TolR residues in the model. The TPH of TolR and three transmembrane helices of TolQ (residues 19–37, 138–156 and 169–187) are shown. The figure highlights the proximity of residues TolQ Thr145, Thr178 and TolR Asp23 within the model, all of which have been identified previously as functionally important (Goemaere *et al*. 2007b). The conserved residue Phe32 is also shown. **E**, Comparison of the electrostatic surfaces for the cytoplasmic chambers of the TolQ model with that of the ExbB structure (PDB code: 6TYI) (Celia *et al*. 2019). Figures were generated using chimera (Jurrus *et al*. 2018). Upper panels are cut-throughs of each stator protein while the lower panels are 90° rotations showing the cytoplasmic constriction. The TolQ chamber is predominantly electronegatively charged whereas ExbB has bands of positive and negative charge. The transmembrane region of both proteins is a predominantly neutral pore in which the TPHs of the TolR dimer reside (not shown in this figure).

In the most recent cryo-EM structures, a 5:2 complex of ExbB-ExbD is observed in which the two transmembrane helices of the ExbD dimer reside within the pore formed by the ExbB pentamers rather than the membrane (Celia *et al*. 2019). We refer to this region of ExbD as the transpore helix (TPH). Although the TPH shows varying degrees of conservation amongst the stators (TolR and MotB are 66% and 19% identical, respectively, to the TPH of ExbD; Fig.   2A) they all possess conserved aspartic acid and phenylalanine residues (Asp23 and Phe32 in TolR). Native mass spectrometry data for the complex ejected directly from the native *E. coli* inner membrane are also consistent with a 5:2 stoichiometry for ExbB-ExbD (Chorev *et al*. 2018). Given these complementary data, the following discussion of Tol-Pal literature assumes TolQ-TolR also forms a 5:2 complex.

A homology model of TolQ-TolR based on 5:2 subunit stoichiometry is shown in Fig. 2B–D. The TolQ pentameric assembly similarly forms a large cytoplasmic chamber but with distinct charge patterning on its inner surface relative to that seen in ExbB (Fig. 2E). ExbB has a band of positive charge in the middle of the chamber followed by a negative band beneath whereas the TolQ chamber is exclusively negatively charged. Whether these differences in electrostatics are physiologically relevant is not known. Notwithstanding these differences, however, the electrostatic charge state of the transmembrane regions of ExbB and TolQ are similarly neutral. A series of mutagenesis studies have identified several residues within TolR and TolQ as functionally important, some presumed to be part of the proton conducting pathway through the complex. These include Asp23 in the TolR TPH and Thr145, Thr178 and Pro187 in the second and third transmembrane helices of TolQ (Cascales, Lloubes and Sturgis 2001; Goemaere, Cascales and Lloubes 2007a; Goemaere *et al*. 2007b; Zhang *et al*. 2009). The model presented in Fig. 2D shows how TolQ Thr145 and Thr178 are in close proximity to TolR Asp23. A similar constellation of residues are found in the ExbB-ExbD and MotA-MotB stator complexes (Braun and Herrmann 2004 ).

Several studies have demonstrated that TolR is dimeric. *In vivo* disulphide crosslinking centred on TolR TPH residues are consistent with the TPH forming a homodimer (Zhang *et al*. 2009). Other *in vivo* studies have demonstrated that the periplasmic domain of TolR is also dimeric but likely to undergo substantial structural changes in response to the PMF and interactions with TolQ (Journet *et al*. 1999; Goemaere *et al*. 2007b). NMR and crystallographic studies of the periplasmic domains from *H. influenzae* and *E. coli* TolR, respectively, both show dimer structures (Parsons, Grishaev and Bax 2008; Wojdyla *et al*. 2015). The homologous proteins ExbD and MotB have also been shown to be dimeric, by DEER spectroscopy and X-ray crystallography, respectively (O'Neill *et al*. 2011; Celia *et al*. 2016).

The structures of *H. influenzae* and *E. coli* TolR periplasmic domains reveal substantially different dimer interfaces suggesting they represent alternative structural states for the protein. The NMR structure of *H. influenzae* TolR was determined using a construct (residues 59–130) in which both the N- and C-termini of the periplasmic domain were truncated. (Fig. 1A). The β-sheets of each monomer contribute to form a deep cleft similar to a baseball mitt (Parsons, Grishaev and Bax 2008). Wojdyla *et al*. (2015) demonstrated that these sequences in the intact *E. coli* periplasmic domain (residues 36–142) form a strand-swapped dimer in which two additional β-strands and α-helix stabilise the dimer interface and obliterate the deep cleft observed in the truncated *H. influenzae* structure (Fig. 1B) (Wojdyla *et al*. 2015). Notwithstanding these additional sequences, the overall fold of the TolR domain is very similar in the two structures except that the subunits are rotated ∼180° relative to each other. The conformation of the strand-swapped *E. coli* dimer is consistent with earlier *in vivo* cysteine crosslinking studies showing that a spontaneous disulphide formed *in vivo* when Tyr117 was substituted for cysteine. The two residues are only close enough to form a disulphide in the full-length *E. coli* structure, but too far apart in the rearranged (truncated) *H. influenzae* structure. Moreover, formation of the disulphide inactivates Tol-Pal *in vivo* and blocks proton transport (Goemaere *et al*. 2007b), consistent with the structural changes associated with the TolR dimer being linked to PMF activation of the stator complex.

TolR binds PG but the molecular details are not yet known. Wojdyla *et al*. (2015) found that only the truncated form of *E. coli* TolR could bind to PG (in the form of isolated sacculi) whereas the full-length, strand-swapped dimer had no PG binding activity (Wojdyla *et al*. 2015; Boags, Samsudin and Khalid 2019). Similar findings have also been reported for MotB (Roujeinikova 2008; O'Neill *et al*. 2011; Kojima *et al*. 2018). The picture emerging is one in which the shortened forms of MotB and TolR (and possibly ExbD, although this has not been demonstrated directly) bind to PG whereas PG binding is inhibited in the full-length proteins, most likely due to the combined effects of conformational rearrangement and occlusion of the PG binding site. The N-terminal sequences of the periplasmic domain in the full-length versions of MotB and TolR are of sufficient length when extended as disordered sequences to allow the PG-binding dimer to reach the cell wall, ∼90 Å from the inner membrane. While the molecular details of PG recognition by these dimers remains to be established, the structure of *H. pylori* MotB bound to the N-acetyl muramic acid (NAM) moiety of PG offers some clues (Roujeinikova 2008). This crystallographic/modelling study suggested glycan chains sit in grooves either side of the dimer interface, with the peptide cross-bridge connecting the two chains bound within the groove although there is no direct evidence for this binding mode.

The available data point to TolR and MotB (and possibly ExbD) dimers existing in either closed or open states. In the closed state (equivalent to the full-length *E. coli* TolR structure; (Wojdyla *et al*. 2015)), the strand-swapped dimer sits close to the surface of the stator partner in the inner membrane which is also thought to close the proton pore of the channel (Goemaere *et al*. 2007b). In the open state, (equivalent to the truncated forms of MotB and TolR; (Parsons, Grishaev and Bax 2008; Roujeinikova 2008), the N-terminal linker residues connecting the periplasmic domains to the TPH helix of each stator unravel enabling the restructuring of the dimer and binding of the cell wall. The PMF activates these large-scale structural transitions by moving protons between the TPH of TolR/ExbD/MotB and the specific inner membrane stator protein partner (TolQ/ExbB/MotA). We note that interatomic distances of residues present in both the TPH dimer and strand-swapped dimer of TolR differ significantly; the distance between Pro37 within these dimers is ∼7 Å and ∼40 Å, respectively. We speculate these differences may reflect changes within the stator that are linked to proton flow and PG binding by the periplasmic domain.

### TolA

TolA is a monomeric 40-kDa inner membrane protein comprised of three domains; a transmembrane helix (TolA^I^), a helical domain rich in alanine and charged residues that is thought to span the periplasm (TolA^II^) and a C-terminal, 12-kDa globular domain (TolA^III^) (Levengood, Beyer and Webster 1991; Witty *et al*. 2002). There are no structures available for intact TolA.

**Figure 3.**
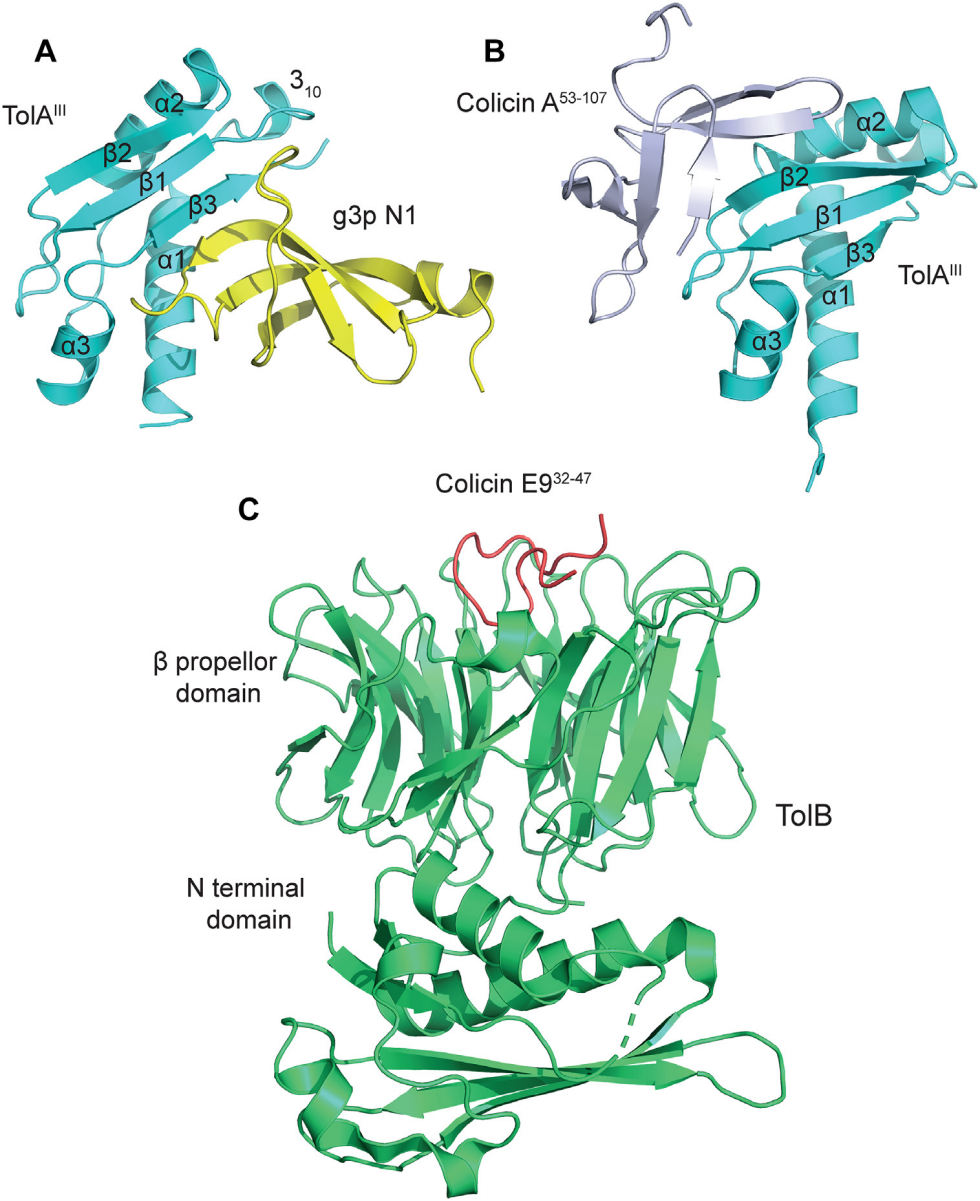
Structural basis for Tol-Pal parasitism by bacteriocins and filamentous bacteriophages. See text Box 1 for details. **A**, Phage g3p N1 domain binds *E. coli* TolA^III^ through a β-strand augmentation mechanism at the same site as TolB (Fig. 1D) but in the opposite orientation (PDB code: 1TOL) (Lubkowski *et al*. 1999). **B**, Colicin A (residues 53–107) also binds TolA^III^ through β-strand augmentation, but on the opposing side of the β-sheet targeted by phage g3p N1 and TolB (PDB code: 3QDR) (Li *et al*. 2012). **C**, Crystal structure of the colicin E9 translocation (T-) domain (residues 32–47) bound to TolB (PDB code: 2IVZ) (Loftus *et al*. 2006). Colicin E9 binds at the same β-propeller site on TolB as used by Pal but does not induce the conformational changes in TolB that sequester its N-terminus, as in Fig. 1F. The N-terminus of TolB in this complex (not shown) is disordered thereby promoting binding to TolA^III^ (Bonsor *et al*. 2009).

Studies from a number of laboratories have shown that TolA is a protein-protein interaction hub, able to form complexes with Tol proteins (Derouiche *et al*. 1995) and CpoB/YbgF in the periplasm (Walburger, Lazdunski and Corda 2002, Krachler *et al*. 2010b) as well as being targeted by bacteriophages and bacteriocins (Cascales *et al*. 2007; Kleanthous 2010), to promote their entry into cells (Fig. 3 and Box 1). Most of these interactions have been validated through structural and biophysical analysis. Other TolA interactions however have proven controversial either for lack of corroborating biochemical data or because they are contradicted by other work. For example, crosslinking studies have implicated TolA^I^ as interacting with both TolQ and TolR, forming a TolQ-TolR-TolA complex in the inner membrane (Germon *et al*. 1998). Yet, as described above, the TolR TPH probably does not reside in the membrane (Celia *et al*. 2016; Celia *et al*. 2019). Similarly, TolA^III^ was shown by crosslinking and immunoprecipitation assays to interact with Pal in the outer membrane (Cascales *et al*. 2000). However, no such interaction is observed by a range of biophysical methods using purified proteins (Bonsor *et al*. 2009). Moreover, Pal residues purportedly involved in binding TolA^III^ (Cascales and Lloubes 2004) in fact form the high affinity binding site for TolB (Bonsor *et al*. 2007; Kleanthous 2010).

**Box 1. Bacteriocins, bacteriophages and Tol-Pal**

Group A colicins and filamentous bacteriophages use protein-protein interactions to hijack the energised Tol-Pal system for entry into *Escherichia coli* cells (Cascales *et al*. 2007; Kleanthous 2010; Atanaskovic and Kleanthous 2019). Group A colicins bind to a specific surface receptor on the target bacterium from where they recruit an outer membrane porin (OmpF or OmpC), the pores of which are used to reach either TolA or TolB in the periplasm (Loftus *et al*. 2006; Housden *et al*. 2010; Housden *et al*. 2013). Filamentous bacteriophages (f1, fd, M13)) use the conjugating F-pilus as their receptor and thereafter target TolA. Some bacteriophages and group B colicins parasitise the Ton system for entry but are not dealt with further here (see (Cascales *et al*. 2007)). Phages appear not to require Tol-Pal be coupled to the proton motive force for cell entry (Samire *et al*. 2020). Involvement of the PMF in colicin translocation remains controversial (Cramer, Sharma and Zakharov 2018). Several studies however clearly point to its requirement in the early stages of import across the outer membrane (Bonsor *et al*. 2009; Vankemmelbeke *et al*. 2009).

Fig. 3 shows the modes of binding of Ff phage coat protein g3p (*a*) and colicin A (*b*) with TolA^III^, and colicin E9 with the β-propeller domain of TolB (*c*). The small N1 domain of g3p interacts with β3 of TolA^III^ through β-strand augmentation (Lubkowski *et al*. 1999), but adopts an anti-parallel orientation compared to the parallel orientation seen in the endogenous TolA^III^-TolB complex (Szczepaniak *et al*. 2020) (compare with Fig. 1D). Colicin A on the other hand binds through an alternative β-strand augmentation site, interacting with β2 on the opposite side of the β-sheet (Fig. 3B) (Li *et al*. 2012). Bacteriocins such as colicins E2-E9 target TolB using an intrinsically disordered protein epitope that is part of the bacteriocin's translocation domain (residues 32–47) (Bonsor *et al*. 2009). Colicin E9 mimics interactions of Pal with TolB but without inducing the structural changes in TolB that normally diminish its binding to TolA (Loftus *et al*. 2006; Bonsor *et al*. 2009). This stealth mechanism enables the surface-bound colicin E9 to connect itself to the PMF, which is required for the early stages of import (Rassam *et al*. 2018).

Both TolA^I^ and the equivalent transmembrane region of TonB have a conserved Ser-His-Leu-Ser motif (Koebnik 1993). Germon *et al*. (1998) found that mutating Ser18 and His22 in TolA^I^ diminished TolQ binding (as determined by formaldehyde cross-linking and immunoblotting). Suppressor analysis has identified the first transmembrane helix of TolQ as the likely interaction site for the TolA^I^ motif (Germon *et al*. 1998).

One of the most important interactions of TolA is with the N-terminus of TolB in the periplasm. Suppressor mutation analysis and yeast two hybrid screens originally showed TolA^III^ binds to the N-terminal domain of TolB (Lazzaroni, Dubuisson and Vianney 2002; Walburger, Lazdunski and Corda 2002). The interaction site on TolB was established definitively by deletion and biochemical analysis; removal of the N-terminal 12-amino acids of TolB generates a *tol* phenotype, inhibits binding of TolB to TolA^III^ (Bonsor *et al*. 2009) and abolishes accumulation of Pal at division sites (Szczepaniak *et al*. 2020). Biophysical studies have shown that the TolA^III^-TolB complex has a low affinity (K_d_∼40–200 μM, depending on the species), a consequence of the structural rearrangements in TolA^III^. The recent solution state structure for *Pseudomonas aeruginosa* TolA^III^ bound to a TolB peptide shows a β-strand augmentation binding mechanism; the C-terminal helix of TolA^III^ is displaced by the N-terminal residues of TolB, which form a parallel β-strand (Szczepaniak *et al*. 2020) (Fig. 1D). The resulting complex is structurally similar to that of TonB bound to the TonB box of TonB dependent transporters (TBDTs) in the outer membrane (2.2 Å rmsd). The architecture of TonB-TBDT complexes, which can also have similarly low affinities, makes them mechanically stable (Chen, Radford and Brockwell 2015; Hickman *et al*. 2017). The role of the PMF-coupled ExbB-ExbD stator complex is to exploit this mechanical stability to dislodge the plug domains of ligand-bound TBDTs via their complexes with TonB. We suggest that TolA through its coupling with the PMF-linked TolQ-TolR stator complex adopts a similar role, but one in which TolB is dislodged from its complex with Pal at the outer membrane via a TolA-TolB-Pal ternary complex (Szczepaniak *et al*. 2020). The biological rationale for energised dissociation of the TolB-Pal complex is explored below.

There is no structural information available for the central 25-kDa domain of TolA (TolA^II^), > 50% of which is alanine, lysine and glutamate; the motif Lys-Glu–Ala_3_–Glu/Asp is repeated thirteen times (Levengood, Beyer and Webster 1991; Derouiche *et al*. 1995; Schendel *et al*. 1997). Solution X-ray scattering and far-UV circular dichroism predict that TolA^II^ has an elongated helical structure, possibly involving a three-helix bundle (Derouiche *et al*. 1999; Witty *et al*. 2002). Several pieces of evidence shed light on how TolA^II^ might function. First, deletion analysis suggests the length of the domain is important (Schendel *et al*. 1997). Second, TolA undergoes structural changes in response to the PMF although the details of these rearrangements are obscure (Germon *et al*. 2001). Third, fluorescence recovery after photobleaching (FRAP) and single particle tracking (SPT) data show that GFP-TolA displays unrestricted Brownian motion in the inner membrane of non-dividing cells (Rassam *et al*. 2018) prior to its recruitment to the divisome (Gerding *et al*. 2007; Rassam *et al*. 2018). Fourth, microscopy data suggest TolA can fully extend through the periplasm, as demonstrated by the capture and restriction of GFP-TolA in the inner membrane by colicin-bound TolB at the outer membrane (Rassam *et al*. 2018). Cumulatively, these data suggest that while TolA^II^ can extend through the PG layer to reach the outer membrane it cannot be permanently extended as this would entrap TolA in the holes that exist in the PG (Turner *et al*. 2013) – estimated to be ∼50–100 Å diameter (Turner *et al*. 2013) – restricting its diffusion.

We speculate that cycles of TolA^II^ extension and retraction are linked to proton flux through the TolQ-TolR stator complex. Extension-retraction may also be a feature of TonB activity. A number of studies have shown that TBDT ligands activate transcription of their respective TBDT gene through a specific sigma factor-anti-sigma factor regulatory complex at the inner membrane (Noinaj *et al*. 2010). For example, the N-terminal periplasmic domain of the ferric citrate TBDT FecA interacts with the C-terminal periplasmic domain of the FecR regulator in the inner membrane, activating transcription of *fecABCDE* transport genes (Enz *et al*. 2003). Given the similarities between the Ton and Tol-Pal systems we speculate that such signalling might be based on TonB pulling the TonB box of a TBDT through the PG layer so its associated N-terminal signalling domain can physically interact with transcriptional regulators in the inner membrane. Below we explore how extension-retraction of TolA could be linked to the outer membrane stabilising role of Tol-Pal.

### TolB

TolB is a 45-kDa soluble periplasmic protein that is also an interaction hub. The structure of *E. coli* TolB (Abergel *et al*. 1999; Carr *et al*. 2000) (Fig. 1E) and TolB in complex with Pal (Bonsor *et al*. 2007; Bonsor *et al*. 2009) (Fig. 1F), TolA (Szczepaniak *et al*. 2020), colicin A (Zhang *et al*. 2010) (note, colicin A binds to both TolA and TolB) and colicin E9 (Loftus *et al*. 2006) (Fig. 3C) have all been reported. Other interaction partners have also been identified *in vivo* (including Lpp and OmpA) (Clavel *et al*. 1998), but these have not been validated *in vitro* nor structurally characterised. TolB is composed of two distinct domains; an N terminal α/β domain which binds TolA and a C-terminal, six-bladed β-propeller domain that binds Pal (Fig. 1F). The two domains are connected by a 9-residue linker sequence. The different structures of TolB combined with biophysical studies demonstrate that the protein is in conformational equilibrium, its different states favoured by specific binding partners. Pal stabilises largescale structural changes in TolB relative to the unbound state in which TolB's N-terminus becomes sequestered between its two domains (Bonsor *et al*. 2007; Bonsor *et al*. 2009) (Fig. 1F). Consequently, Pal diminishes TolB's interaction with TolA since the TolB N-terminus constitutes the TolA binding site. Conformational changes in TolB's two domains ensue when the protein dissociates from Pal, releasing its N-terminus from its interdomain binding site and promoting binding to TolA.

Using *in vitro* chemical crosslinking, Bonsor *et al*. (2009) also found a third, lowly-populated (presumably high energy) conformational state involving a ternary TolA-TolB-Pal complex (Bonsor *et al*. 2009). In this Pal-bound state, the N-terminus of TolB becomes dislodged from its interdomain binding site, enabling binding to TolA. Although not understood at the time the ternary complex likely plays a central role in the postulated force-dependent dissociation of the TolB-Pal complex (see below). Steered molecular dynamics simulations suggest the force required to dissociate the TolB-Pal complex is greater when the N-terminus of TolB is bound between its two domains (Szczepaniak *et al*. 2020). These simulations are consistent with the need for TolB's N-terminus to become dislodged from the body of TolB in the Tol-Pal complex to enable force-dependent dissociation. Moreover, they reveal that several conserved TolB linker residues mediate communication between TolB's N-terminal domain, where force *in vivo* is presumably applied, and the C-terminal β-propeller domain where Pal is bound. Mutation of these residues generates *tol*-like phenotypes consistent with such a role *in vivo* (Szczepaniak *et al.*^2020^).

### Pal

Pal (peptidoglycan associated lipoprotein) is attached to the inner leaflet of the outer membrane by an N-terminal lipid anchor from where it binds either PG (Lazzaroni and Portalier 1992) or TolB (Bouveret *et al*. 1995; Clavel *et al*. 1998). Both crystal and NMR structures of Pal have been reported (Abergel *et al*. 2001), the latter bound to a fragment of PG (Parsons, Lin and Orban 2006). Pal has an α/β sandwich fold (Fig. 1G), the loops connecting its elements of secondary structure comprising the PG-binding site (Fig. 1H). Pal is a member of the same large family of PG-binding proteins that includes TolR and MotB but in contrast to these proteins is monomeric. Pal binds the diaminopimelic acid residue (mDAP) of non-crosslinked stem peptides within PG, utilising conserved aspartic acid and arginine residues (Asp71 and Arg73 in *H. influenzae* Pal) (Parsons, Lin and Orban 2006).

One of the consequences of Pal being simultaneously tethered to the outer membrane and bound to the PG layer is that its lateral diffusion is severely restricted (Szczepaniak *et al*. 2020). Yet a key aspect of Tol-Pal function is the accumulation of Pal at division sites during cell division, showing that the protein is nevertheless mobile on the timescale of cell growth and division (Gerding *et al*. 2007; Petiti *et al*. 2019; Szczepaniak *et al*. 2020). Pal mutations or deletions that inhibit PG binding lead to faster and unrestricted diffusion in the outer membrane but also block outer membrane stabilisation and prevent the protein's accumulation at division sites (Petiti *et al*. 2019; Szczepaniak *et al*. 2020). Pal employs the same residues to bind TolB as are used to bind PG (Bonsor *et al*. 2007). TolB is therefore key to Pal's accumulation at division sites where its role is two-fold; to block Pal binding to PG, thereby increasing its mobility in the outer membrane, and to render the complex a target for force-mediated dissociation by PMF-linked TolQ-TolR-TolA in the inner membrane (Szczepaniak *et al*. 2020).

## MOBILISATION-AND-CAPTURE OF Pal BY Tol PROTEINS USES CELLULAR ENERGY TO INVAGINATE THE OUTER MEMBRANE AT DIVISION SITES

The pleiotropic nature of the *tol-pal* phenotype has confounded efforts to determine the physiological role of Tol-Pal in bacteria since discovery of the *tol-pal* genes. Some involvement in outer membrane stabilisation has always been envisaged but its nature was obscure. In addition, *tol-pal* genes are not essential in some Gram-negative bacteria, which is counter intuitive if the system is required for outer membrane stabilisation. With hindsight, the outer membrane blebbing frequently observed at mid-cell positions of dividing *tol-pal* mutants was an important clue (Weigand and Rothfield 1976; Weigand, Vinci and Rothfield 1976; Fung, MacAlister and Rothfield 1978; Fung *et al*. 1980), which implied that the outer membrane at the constriction zone was dissociating from the cell wall. It was not until 2007 however, when it was demonstrated that all Tol-Pal proteins are recruited to the divisome (Gerding *et al*. 2007), that a role in outer membrane invagination at septation sites seemed likely. This role was originally thought to be that of an energised tether between TolA in the inner membrane and Pal in the outer membrane based on earlier *in vivo* crosslinking data (Cascales *et al*. 2000). However, such a mechanism is unlikely for three reasons. First, as described above, TolA^III^ and Pal do not interact *in vitro*. Second, a direct TolA^III^-Pal interaction obviates the need for TolB in the periplasm yet deletion of *tolB* results in a classic *tol* phenotype. Indeed, mutations in TolB tend to be the most deleterious of all *tol* mutations in *E. coli* (Szczepaniak *et al*. 2020). Third, the TolA^III^-TolB complex is clearly the focal point of the force that is generated by the PMF-linked TolQ-TolR stator complex (Szczepaniak *et al*. 2020). For TolB, a soluble protein, to be the target of force transduction in the periplasm only makes biological sense when viewed in the context of TolB's association with Pal in the outer membrane. This in turn implies that force-mediated dissociation of TolB-Pal *in vivo* must occur via a ternary TolA-TolB-Pal complex so that Pal can (re)bind PG.

Which brings us to the recent studies of (Petiti *et al*. 2019) and (Szczepaniak *et al*. 2020). Both studies demonstrate that a major physiological role of the entire Tol-Pal assembly is the PMF-driven accumulation of Pal at division sites, where its binding of the cell wall helps invaginate the outer membrane and prevent blebbing. In addition, Szczepaniak *et al*. (2020) exploited a novel mathematical approach, developed by Seán Murray (Max Planck, Marburg), called SpatialFRAP in order to dissect the underlying mobilisation-and-capture mechanism (Szczepaniak *et al*. 2020). SpatialFRAP was used to extract effective diffusion coefficients (D_eff_) from fluorescence recovery after photobleaching (FRAP) data for Pal-mCherry expressed from the chromosomal locus in *E. coli*. This development was important because the diffusion of Pal varies both spatially and temporally during the *E. coli* cell cycle. Consequently, FRAP curves do not plateau and so standard FRAP analyses cannot be used to determine diffusion coefficients. Employing SpatialFRAP in conjunction with engineered strains Szczepaniak *et al*. (2020) uncovered the definitive characteristics of Pal mobility and the role of Tol proteins (Szczepaniak *et al*. 2020). First, the mobility of Pal in the outer membrane of non-dividing cells is very slow (effective diffusion coefficient, D_eff_ ∼10^−4^ µm^2^.s^−1^) due to binding of the PG. Second, the onset of division leads to an acceleration in Pal mobility throughout the cell except at the division site where instead Pal molecules accumulate and mobility is similar to that in non-dividing cells. Third, all components of the Tol system and the PMF are required for these combined effects. A particularly remarkable aspect of this mechanism is the action-at-a-distance on Pal mobility when the divisome is formed. How do Pal molecules far from the divisome have their outer membrane mobility enhanced while those at the divisome do not and how is cellular energy expended to achieve these joint outcomes?

**Figure 4.**
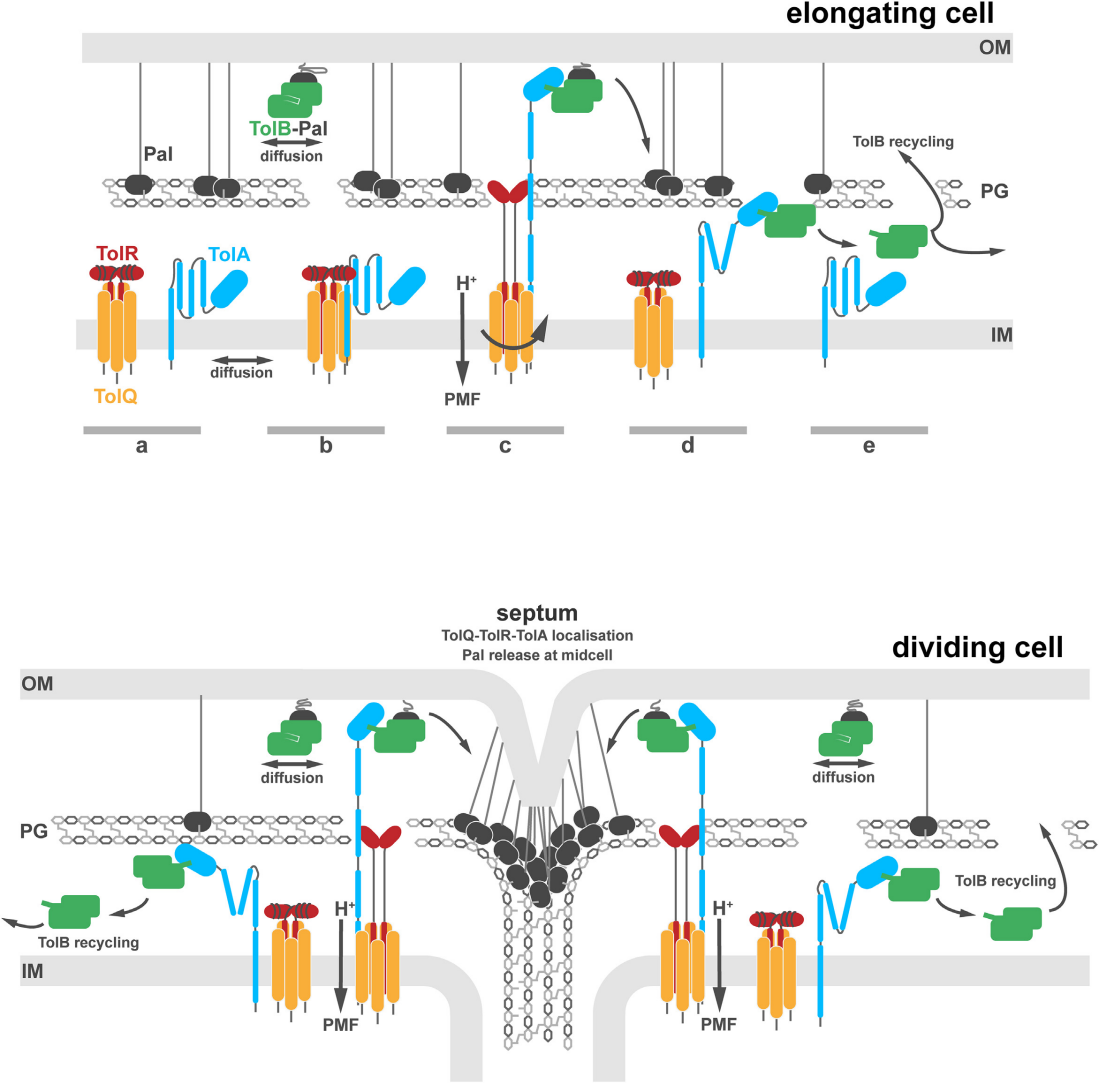
PMF-driven mobilisation-and-capture of Pal by Tol proteins drives Pal accumulation at division sites. Figure adapted from (Szczepaniak *et al*. 2020). See text for details. The following model assumes that TolB-Pal complexes are actively dissociated by PMF-linked TolQ-TolR-TolA, and that dissociated TolB molecules are translocated through holes in the PG layer by TolA. *Top panel—Elongating cell*. **A**, The stator complex TolQ-TolR (depicted as a 5:2 complex based on the modelling presented in Fig. 2) and TolA are free to diffuse in the inner membrane (IM). The periplasmic domain of TolR is shown as a strand-swapped dimer, consistent with available structural data (Fig. 1). Pal is bound to the mDAP moiety of peptidoglycan (PG; white line against grey Pal) unless in complex with TolB, which blocks PG binding and increases Pal diffusion in the outer membrane (OM). **B**, TolA associates with TolQ-TolR. It is not known if the complex is a stable TolQ-TolR-TolA complex or if the association is transient. **C**, Proton flux through the residues of the TolQ pentamer and the transpore helices of the TolR dimer, coupled to possible rotatory motions of the stator subunits, cause unravelling of the strand-swapped periplasmic domain of TolR allowing it to extend and bind the cell wall. Consequent with these changes, TolA extends through a hole in the PG layer, possibly aided by interactions with the TolR-PG complex. At the outer membrane, TolA binds the N-terminus of TolB which is in complex with Pal. **D**, Loss of protonation causes the whole assembly to relax back to its starting position, providing the driving force to bring TolB down through the PG layer into the lower periplasmic compartment. **E**, TolB now dissociates from TolA—presumably because TolA is no longer exerting a force and the complex has a weak affinity—and diffuses until it encounters a hole in the PG through which it can reach the outer membrane and rebind Pal to repeat the process. *Bottom panel—Dividing cell*. The TolQ-TolR-TolA complex is recruited to the divisome which confines its TolB capturing activity. As TolB-Pal complexes diffuse past the septum they are actively dissociated, releasing Pal and recycling TolB, as described above. Thus, Pal located at the divisome is kept free of TolB by localised TolQ-TolR-TolA. Recycled TolB diffuses away and mobilises non-septal Pal molecules. Because TolQ-TolR-TolA is not freely circulating this leads to a greater number of TolB molecules being located in the outer periplasmic compartment (i.e. TolB-Pal complexes are longer lived than in an elongating cell) and as a result Pal mobility increases throughout the cell except at the septum. More and more Pal molecules now accumulate at the septum where they stabilise the link between the outer membrane and the underlying cell wall in daughter cells.

The answer as we currently understand it is comprised of four elements, two reasonably well-understood and two hypothetical (Fig. 4). The well-established elements are: (1) the recruitment of PMF-linked TolQ-TolR and TolA to the divisome, albeit the mechanism is still not known (Gerding *et al*. 2007; Petiti *et al*. 2019), and, (2) TolB's inhibition of PG binding by Pal (Bonsor *et al*. 2009), which likely increases Pal mobility in the outer membrane and the chances of a diffusing TolB-Pal complex being captured by TolQ-TolR-TolA at the divisome. The two elements for which there is as yet no direct evidence are: (1) active dissociation of TolB-Pal complexes by PMF-linked TolQ-TolR-TolA, and (2) translocation of dissociated TolB molecules through holes in the PG layer by TolA, the same holes TolA itself would have used to reach the outer membrane in the first place (Fig. 4). We argue that it is this spatial separation of TolB molecules (those actively dissociated by TolQ-TolR-TolA from those remaining bound to Pal in the outer membrane) by the intervening PG layer that explains action-at-a-distance on Pal mobility. Because of this spatial separation, dissociated TolB molecules can only diffuse between the inner membrane and the PG until a hole is found through which they can again reach the outer membrane to rebind Pal (Fig. 4). We note that this is not the first model to suggest the importance of PG pores for spatial separation of periplasmic proteins. Regulation of peptidoglycan synthesis, for example, involves outer membrane lipoproteins reaching through holes in PG to interact with inner membrane proteins, as in the case of LpoB and PBP1B (Egan and Vollmer 2013; Turner *et al*. 2013; Egan *et al*. 2017). Sacculi are known to contain pores as large as 5–16 nm (Turner *et al*. 2013), which presumably also reflects the situation at the septum where largescale remodelling takes place during cell division. To conclude, TolB serves as a PMF-recycled catalyst of Pal mobility, mobilising Pal molecules anywhere in the cell except at the divisome where Pal is kept free of TolB through the localised action of TolQ-TolR-TolA.

A major change in Pal mobility ensues in non-dividing cells when the TolQ-TolR-TolA complex is no longer confined to the divisome (Szczepaniak *et al*. 2020). Now, a TolB-Pal complex anywhere in the cell can be captured by diffusing TolQ-TolR-TolA, releasing Pal to bind the cell wall. The net result is that in non-dividing cells Pal is predominantly bound to the PG because the small number of TolB molecules (present at ∼10% the levels of Pal) that could increase its diffusion are prevented from doing so. As a result, Pal's lateral diffusion in the outer membrane slows. A potential consequence of TolB-Pal complexes being continually captured by TolQ-TolR-TolA in non-dividing cells is the redistribution of Pal in the cell envelope, which is further addressed below.

How is the mobilisation-and-capture of Pal described above linked to force generation by TolQ-TolR-TolA? We speculate that extension-retraction of TolA may be coincident with the flow of protons through the stator and the (as yet unresolved) structural changes in TolQ that cause unfurling of the strand-swapped TolR dimer so that it can extend and bind PG (Fig. 4). In this TolR-PG-anchored state, TolA extends through the periplasm, possibly also interacting with PG-bound TolR (a similar interaction has been suggested to occur between ExbD and TonB; (Ollis and Postle 2012)), to capture TolB from a TolB-Pal complex in the outer membrane. Reversal of these steps, for example through the loss of protonation, would result in both TolR and TolA, the latter now bound to TolB, returning to the inner membrane in their retracted states. We suggest that the TolQ-TolR-TolA complex may be continuously going through this cycle in response to the PMF.

The mechanism we propose for the Tol-Pal system raises many questions that are also pertinent for other PMF-driven nanomachines in the bacterial cell envelope. Does the movement of protons through these conserved complexes transduce force to their specific partners, the flagellum, TonB, TolA, by similar mechanisms? In the case of the bacterial flagellum, many MotA-MotB stators engage with the flagellum and even exchange during active rotation of the flagellum (Leake *et al*. 2006; Reid *et al*. 2006; Brenzinger *et al*. 2016). How many stators are involved in driving the motion of TonB and TolA? Alternatively, can more than one TonB/TolA engage with a single stator complex? What are the structural transitions experienced by TonB and TolA and how are these coupled to the unplugging of TBDTs and the dissociation of TolB-Pal complexes, respectively, in the outer membrane? Can the plug domains of TBDTs bound to TonB be brought through the PG layer as we have postulated for TolB bound to TolA?

## Tol-Pal INVOLVEMENT IN REMODELLING SEPTAL PEPTIDOGLYCAN AT DIVISION SITES

Recent work has revealed that once localised to the divisome, the Tol-Pal assembly has a broader role within the cell envelope beyond stabilising the connection between the outer membrane and cell wall. Tol-Pal is also involved in remodelling the PG at division sites. One of these roles involves *cpoB*, the terminal gene in the *tol-pal* operon. CpoB (coordinator of PG synthesis and outer membrane constriction associated with PBP1B, formerly known as YbgF) is a 28 kDa periplasmic protein that has long been an enigma. Although widely conserved in bacteria, deletion of *cpoB* does not generate a characteristic *tol-pal* phenotype but does sensitise cells to certain β-lactam antibiotics, such as cefsulodin, which target penicillin binding protein 1B (PBP1B). Krachler *et al*. (2010b) demonstrated that CpoB has an elongated oligomeric structure, composed of a trimeric coiled-coil attached to a three-repeat tetratricopeptide repeat (TPR) domain, and that this structure is disrupted when the TPRs of CpoB associate with TolA^II^, generating a heterodimeric CpoB-TolA complex (Krachler, Sharma and Kleanthous 2010a; Krachler *et al*. 2010b). Subsequent studies by Gray *et al*. (2015) showed that CpoB is an important regulator of PBP1B transpeptidase activity and that this regulation is further moderated by PMF-linked TolQ-TolR-TolA (Gray *et al*. 2015).

PBP1B is an inner membrane bifunctional PG synthase with both glycosyltransferase and transpeptidase activity. These activities are stimulated by the outer membrane lipoprotein LpoB, resulting in PBP1B producing hyper-crosslinked PG. The TPR domain of CpoB associates with PBP1B to block LpoB-mediated activation of PG crosslinking thereby generating fewer peptide crosslinks within the PG. TolA, which also binds to PBP1B, reverses the inhibitory effect of CpoB on PBP1B transpeptidase activity, reinstating hyper-crosslinked PG. It is not clear what the oligomeric status of CpoB is when bound to PBP1B nor if the same (or different) regions of the CpoB TPR domain that bind TolA also bind PBP1B. Importantly, however, TolA needs to be coupled to the PMF, which implies that in order for CpoB's inhibitory effect on PBP1B-LpoB transpeptidase activity to be reversed TolA^II^ must extend through the PG layer. Hence, not only is the PMF-linked Tol-Pal system involved in loading division septa with Pal that bind non-crosslinked stem peptides within PG, but it also regulates the degree of peptide crosslinking at these sites by modulating CpoB's influence on PBP1B-LpoB transpeptidase activity.

In the latter stages of bacterial cell division glycan strands connecting daughter cells need to be cleanly cut. Two recent studies point to Tol-Pal being involved in this process. During daughter cell separation crosslinks connecting glycan strands are cut by amidases and endopeptidases. Their action is tightly controlled by specific activators, NlpI (Banzhaf *et al*. 2020) and NlpD and EnvC (Uehara *et al*. 2010). Tol-Pal exerts a degree of control over amidase activity through NlpD. Although no direct interactions between Tol proteins and NlpD have been described, cells deficient in *envC* and *tol* genes display the same growth defects as cells lacking both amidase regulators (Tsang, Yakhnina and Bernhardt 2017) suggesting Tol-Pal may be involved.

One of the phenotypic outcomes of *tol-pal* mutations is cell chaining (Fung, MacAlister and Rothfield 1978; Fung *et al*. 1980; Gerding *et al*. 2007). Given the importance of Tol-Pal for invaginating the outer membrane this phenotype has always been interpreted as demonstrating an outer membrane defect in *tol-pal* mutants. Yakhnina and Bernhardt (2020) reported recently that this is not the case (Yakhnina and Bernhardt 2020). Instead, Tol-Pal is needed for efficient processing of septal PG. They found that sacculi generated from *tol-pal* mutants are also chained suggesting that the Tol-Pal system plays a role in promoting the cleavage of PG-linked daughter cells. Yakhnina and Bernhardt (2020) conducted a phenotypic suppressor screen to identify cell components involved in this activity (Yakhnina and Bernhardt 2020). They identified a number of suppressors in the protease Prc which, together with its partner protein NlpI, hydrolyses the cell wall endopeptidase MepS. Subsequent multicopy suppressor analysis identified other PG hydrolase targets of Prc, including a novel amidase christened DigH, the overexpression of which complemented the cell chaining phenotype of *tol-pal* mutants. These authors also showed that DigH is recruited to the divisome independent of Tol-Pal and that it preferentially cleaves glycan chains lacking stem peptides. These observations may help explain why in some species of bacteria (for example *Chlamydia* spp) a lytic transglycosylase is associated with the *tol-pal* operon that could serve a similar role in cleaving glycans connecting daughter cells. How Tol-Pal promotes efficient septal PG hydrolysis via DigH and other lytic transglycosylases and whether this requires the PMF remains to be established.

## Tol-Pal AND PHOSPHOLIPID TRAFFICKING

*E. coli tol-pal* mutants accumulate phospholipids in their outer membranes, similar to *bam* and *lptD* mutants (Shrivastava, Jiang and Chng 2017). However, unlike *bam* mutants, *tol-pal* mutants seem to have impaired retrograde phospholipid transfer to the inner membrane and retain phospholipids in the outer leaflet of the outer membrane (Shrivastava, Jiang and Chng 2017; Shrivastava and Chng 2019). Unidirectional transport of phospholipids in and out of the outer membrane is mediated by the Mla system, mutations in which affect the lipid asymmetry of the outer membrane (Malinverni and Silhavy 2009). Overexpression of *mla* in *tol-pal* cells partially recovers outer membrane asymmetry (Shrivastava, Jiang and Chng 2017). It has been suggested that since the same phospholipid-retaining phenotype is observed in conditions where *tol* cells are able to divide normally (Gerding *et al*. 2007) then this phenotype does not stem from cell septation problems (Shrivastava, Jiang and Chng 2017). However, it is possible that phospholipids are retained in the outer leaflet of the outer membrane to compensate for the loss of the Tol-Pal system. Another Tol-Pal connection to phospholipid biosynthesis is the *tol-pal* operon gene *ybgC*, which encodes a thiol diesterase (Gully and Bouveret 2006). In *S*. Typhimurium, *ybgC* mutants accumulate phosphatidylglycerol and phosphatidylethanolamine in the outer membrane, similar to *tol-pal* mutants (Masilamani, Cian and Dalebroux 2018). There is increasing evidence that MCE transporter proteins such as LetB (Isom *et al*. 2020) form protein tunnels that act as conduits for phospholipids to the outer membrane, further suggesting that any involvement in phospholipid trafficking by Tol-Pal is indirectly linked to its outer membrane stabilising role.

## Tol-Pal AND POLAR LOCALIZATION OF PROTEINS

The Tol-Pal system has been implicated in the polar localization of several inner membrane proteins with consequent impact on bacterial development and behaviour. In *C. crescentus* the system is required for polar localization of TipN, which regulates cell asymmetry and polar development in the organism (Yeh *et al*. 2010). Tol-Pal is required for cell motility in both *P. putida* and *E. coli* (Llamas, Ramos and Rodriguez-Herva 2000; Youderian *et al*. 2003; Gao, Meng and Gao 2017). In *E. coli* this has been shown to be due to recruitment of chemoreceptor clusters to cell poles (Santos *et al*. 2014; Neeli-Venkata *et al*. 2016). Although co-immunoprecipitation analyses in these studies show that Tol-Pal proteins associate with the proteins being localized to the poles it remains to be established if this is due to direct interactions with Tol-Pal proteins or an indirect result of Tol-Pal activity; for example, the accumulation of Pal at new poles following the completion of cell division. It is also not known if Tol-Pal coupling to the PMF is required for polar localization of these systems.

## WIDER IMPLICATIONS AND FUTURE PERSPECTIVES

Why do bacteria need an energised system to stabilise the connection between the outer membrane and the underlying cell wall when other PG binding proteins exist in the outer membrane that could conceivably carry out the same stabilising function? In *E. coli*, the two other main PG binding proteins in the outer membrane are OmpA and Braun's lipoprotein, Lpp. OmpA, which has a similar abundance to Pal in the outer membrane (∼10^5^ copies), is composed of an integral outer membrane β-barrel and a periplasmic PG binding domain similar to that of Pal. The biogenesis of outer membrane proteins can occur everywhere except the poles in *E. coli* (Rassam *et al*. 2015) and so OmpA could contribute to outer membrane stabilisation at the divisome. However, only those molecules inserted close to the divisome would be useful in this regard since OmpA cannot diffuse laterally in the outer membrane (Verhoeven, Dogterom and den Blaauwen 2013).

Lpp, one of the most abundant proteins in bacteria (∼10^6^ copies), is covalently cross-linked by a suite of transpeptidases to the same mDAP side-chain to which Pal binds non-covalently (Asmar and Collet 2018). *lpp* mutants, which also have destabilised outer membranes, can be rescued by overexpressing *pal* but *pal* mutants are not similarly rescued by *lpp* overexpression (Cascales *et al*. 2002). This observation demonstrates that Lpp cannot compensate for the loss of Pal's outer membrane stabilising function at division sites whereas Pal can compensate for the loss of Lpp crosslinks, although it has been reported that *tol* mutants have less Lpp bound to PG (Weigand and Rothfield 1976). Since Pal actively relocates to mid-cell *en masse* through the action of the Tol-Pal system and its binding is mutually exclusive with the covalent attachment of Lpp we speculate that it may have a role in modulating Lpp crosslinking to PG at division sites. When viewed in the context of Tol-Pal's regulation of PG peptide crosslinking density at division sites (via CpoB) and its involvement (direct or indirect) in the cleavage of glycan chains, these observations all point to Tol-Pal being able to coordinate outer membrane invagination with separation of daughter cells. Moreover, the status of this coordinating role is communicated to the FtsZ constriction ring in the cytoplasm since a delay in the recruitment of Tol-Pal to the divisome delays closure of the Z-ring (Rassam *et al*. 2018).

Why is the *tol-pal* operon essential in some Gram-negative bacteria but not others? In some instances, the answer may lie in the lack of redundancy in systems that stabilise the outer membrane; *C. crescentus*, for example, where *tol-pal* is essential, lacks *lpp* (Yeh *et al*. 2010). Environmental factors could also contribute to *tol-pal*’s importance especially if these place additional stresses on the outer membrane; for example, *tol-pal* is essential for the infection of hosts by many pathogens (Bowe *et al*. 1998; Fortney *et al*. 2000; Dubuisson *et al*. 2005; Abdelhamed *et al*. 2016; Masilamani, Cian and Dalebroux 2018; Hirakawa *et al*. 2019). A major factor in the successful exploitation of diverse ecological niches by Gram-negative bacteria is the presence of O-antigen in the outer membrane which brings additional stability to the membrane. Species where *tol-pal* is essential such as *P. aeruginosa* present O-linked sugars on the surface (Rivera *et al*. 1988) whereas *E. coli* K-12, where *tol-pal* is not essential, does not produce O-antigen. Might the presence of O-antigen on the bacterial surface require cells to have *tol-pal*? The study of Gaspar *et al*. (2000) suggests this might be the case (Gaspar *et al*. 2000). This study asked two related questions: Is *tol-pal* essential in wild-type *E. coli* O7 antigen expressing strains and what happens when O7 antigen expression is introduced into *E. coli* K-12 cells which otherwise does not make O7? They found that *tol-pal* genes could not be deleted from *E. coli* O7 and that O7 expression in *E. coli* K-12 was significantly reduced if these strains also carried *tol-pal* deletions. *tol-pal* essentiality may therefore stem from problems invaginating the outer membranes of soon-to-be-daughter cells if these are inter-digitated due to the presence of O-antigen. Such inter-digitation might require an energised system to invaginate the outer membrane and so separate cells, whereas this requirement might be relaxed in the absence of O-antigen. Consistent with this idea, coarse-grained molecular dynamics simulations of asymmetric outer membrane models indicate that the strong cohesive interactions of tightly-packed O-antigen in smooth LPS make the membrane much more resistant to mechanical deformation compared to rough LPS that lacks O-antigen (Jefferies, Shearer and Khalid 2019). There are exceptions however that contradict this idea. *Salmonella enterica* serovar Typhimurium is able to produce full-length LPS in a *tol-pal* background (Prouty, Van Velkinburgh and Gunn 2002) albeit these mutants decrease the LPS content of the outer leaflet by retaining phospholipid (Masilamani, Cian and Dalebroux 2018).

The TolQ-TolR-TolA complex is released from the divisome when septation is complete, leaving the proteins free to diffuse in the inner membrane. Pal that had accumulated at the divisome is polar in daughter cells redistributes before the next division (Szczepaniak and Kleanthous, unpublished observations). This redistribution is also likely to be dependent on TolQ-TolR-TolA and the PMF since Pal diffusion is too slow otherwise. If this is the case, this would imply that even when diffusing in the inner membrane the TolQ-TolR-TolA complex uses the PMF to scan the outer membrane for TolB-Pal complexes on which to pull (as postulated in Fig. 4). Hence, the Tol-Pal system may have another outer membrane stabilising role in bacteria beyond that at the divisome, as a *de facto* outer membrane surveillance system, using the PMF to redistribute Pal connections to the PG. Such an activity would be advantageous for a Gram-negative bacterium since it could help maintain outer membrane stability in the event of damage, for example, by antimicrobial peptides. An outer membrane surveillance role might be a contributory factor in the pleiotropic instability phenotype typically associated with *tol-pal* mutations, such as the production of OMVs. OMVs mediate macromolecule transfer between bacterial cells and are implicated in biofilm formation and pathogenesis, but how their production is regulated is poorly understood (Schwechheimer and Kuehn 2015). Tol-Pal has long been associated with OMV production since *tol-pal* mutations hyper-vesiculate, particularly at division sites, but it has been unclear if this activity is regulated in any way. The mobilisation-and-capture mechanism uncovered for Tol-Pal could be amenable to regulated production of OMVs through, for example, the modulation of TolB interactions with Pal and/or TolA.

Fig. 3 shows the modes of binding of Ff phage coat protein g3p (*a*) and colicin A (*b*) with TolA^III^, and colicin E9 with the β-propeller domain of TolB (*c*). The small N1 domain of g3p interacts with β3 of TolA^III^ through β-strand augmentation (Lubkowski *et al*. 1999), but adopts an anti-parallel orientation compared to the parallel orientation seen in the endogenous TolA^III^-TolB complex (Szczepaniak *et al*. 2020) (compare with Fig. 1D). Colicin A on the other hand binds through an alternative β-strand augmentation site, interacting with β2 on the opposite side of the β-sheet (Fig. 3B) (Li *et al*. 2012). Bacteriocins such as colicins E2-E9 target TolB using an intrinsically disordered protein epitope that is part of the bacteriocin's translocation domain (residues 32–47) (Bonsor *et al*. 2009). Colicin E9 mimics interactions of Pal with TolB but without inducing the structural changes in TolB that normally diminish its binding to TolA (Loftus *et al*. 2006; Bonsor *et al*. 2009). This stealth mechanism enables the surface-bound colicin E9 to connect itself to the PMF, which is required for the early stages of import (Rassam *et al*. 2018).
